# Supplementary material for: Gonadal sex patterns p21-induced cellular senescence in mouse and human glioblastoma
Source: Commun Biol. 2022 Aug 2;5:781. doi: 10.1038/s42003-022-03743-9 (PMC9345919; doi:10.1038/s42003-022-03743-9)
Supplement: Supplementary file 2 — Supplementary Information [file 42003_2022_3743_MOESM2_ESM.pdf]

Supplementary Table 1. Molecular characteristics of patient tumors from which primary human GBM lines were derived

Line	Sex	MGMT	Copy Number Gain	Copy Number Loss	Mutations	p53 Immuno-reactivity
B2	Female		*EGFR*		*PTEN*	
B5	Female		*EGFR, BRAF, ALK*			
B18	Female	Methylated				
B30	Male	Unmethylated	Polysomy 7			
B31	Male	Unmethylated	Polysomy 7 with *EGFR* amplification, *ALK*		*ATM, APC*	
B36	Male	Methylated		1p36 deletion/ monosomy 1, monosomy 10, 19q13 deletion	*NF1, PTEN, TERT*	Positive
B49	Female	Unmethylated	*ALK*	Monosomy 10	*PTEN, TP53*	Positive
B51	Female		*EGFR*	10q deletion/ monosomy 10		Positive
B66	Male	Unmethylated	Polysomy 7 without *EGFR* amplification, Polysomy 8	Monosomy 10, 19q deletion	*EGFR, PTEN, TP53*	Positive

Supplementary Table 2. MC5 and FC3 gene lists

**MC5 Genes (17)**	**FC3 Genes (9)**
BIRC5	*AK5*
CCNB1	*AMIGO2*
CCNB2	*CHL1*
CDC20	*FERMT1*
CKS2	*IGFBP2*
EZH2	*PCDHB*
KIF20A	*PLAT*
NEFH	*POSTN*
NEFM	*SDC4*
NES	
NUSAP1	
PBK	
PRC1	
PTTG1	
RRM2	
TOP2A	
TPX2	

Supplementary Table 3. Summary of correlation statistics

Figure Panel	Cell Line	Sex	Variables	Slope Estimate (SE, p value)	Slope Difference F vs M (SE)	Slope Difference p value	Correlation (95% CI, p value)	Correlation Difference p value
4a	Nf1-/- DNp53	Male	SA-β-gal ~ *Cdkn2a*	6.27 (6.68, 0.3562)	-9.60 (7.97)	0.2395	0.36 (-0.19 to 0.73, 0.1931)	0.1840
		Female		-3.33 (4.36, 0.4520)			-0.17 (-0.63 to 0.38, 0.5479)	
4b	Nf1-/- DNp53	Male	SA-β-gal ~ *Cdkn1a*	5.38 (2.40, 0.0335)	1.99 (2.80)	0.4823	0.59 (0.11 to 0.85, 0.0212)	0.3464
		Female		7.38 (1.43, 0.00002)			0.79 (0.46 to 0.93, 0.0005)	
4c	Nf1-/- DNp53	Male	SA-β-gal ~ *Cdkn1a/Cdk2*	4.82 (2.37, 0.0524)	3.38 (2.83)	0.2428	0.53 (0.02 to 0.82, 0.0427)	0.2011
		Female		8.20 (1.55, 0.00002)			0.80 (0.50 to 0.93, 0.0003)	
4e	Nf1-/- DNp53	Male	SA-β-gal ~ p21/Cdk2 (24h)	1.64 (1.23, 0.188)	3.43 (1.81)	0.0627	0.30 (-0.06 to 0.59, 0.0997)	0.33
		Female		5.07 (1.32, 0.0003)			0.52 (0.20 to 0.74, 0.0029)	
4f	Nf1-/- DNp53	Male	SA-β-gal ~ p21/Cdk2 (5d)	1.8 (0.91, 0.053)	3.74 (1.38)	**0.009**	0.40 (0.06 to 0.66, 0.0239)	0.19
		Female		5.54 (1.04, 1.57E-06)			0.65 (0.39 to 0.82, 6.47e-05)	
5c	WT Astrocytes	Male	SA-β-gal ~ *Cdkn2a*	-32.44 (43.16, 0.4647)	56.64 (45.66)	0.2352	-0.27 (-0.77 to 0.43, 0.4441)	0.1418
		Female		24.20 (14.91, 0.1269)			0.52 (-0.29 to 0.90, 0.1843)	
5d	WT Astrocytes	Male	SA-β-gal ~ *Cdkn1a*	5.52 (6.37, 0.4005)	13.82 (9.40)	0.1636	0.28 (-0.43 to 0.77, 0.4410)	0.1839
		Female		19.34 (6.91, 0.0142)			0.79 (0.18 to 0.96, 0.0207)	
5e	WT Astrocytes	Male	SA-β-gal ~ *Cdkn1a/Cdk2*	5.06 (3.43, 0.162)	7.92 (4.73)	0.1163	0.39 (-0.31 to 0.82, 0.2608)	**0.0243**
		Female		12.98 (3.25, 0.0014)			0.94 (0.70 to 0.99, 0.0005)	
5g	WT Astrocytes	Male	SA-β-gal ~ p21	12.01 (9.25, 0.2034)	18.19 (12.05)	0.1409	0.33 (-0.30 to 0.76, 0.2909)	0.2375
		Female		30.19 (7.73, 0.0005)			0.67 (0.37 to 0.85, 0.0003)	
5h	WT Astrocytes	Male	SA-β-gal ~ p21/Cdk2	1.33 (1.91, 0.4935)	2.30 (2.32)	0.3284	0.20 (-0.43 to 0.69, 0.5418)	0.3304
		Female		3.63 (1.31, 0.0092)			0.53 (0.16 to 0.77, 0.0081)	
6c	Human GBM	Male	SA-β-gal ~ p21 (24h)	6.57 (13.08, 0.6247)	8.73 (13.53)	0.5309	0.23 (-0.56 to 0.81, 0.5783)	0.1116
		Female		15.30 (3.49, 0.0009)			0.85 (0.35 to 0.97, 0.0080)	
6d	Human GBM	Male	SA-β-gal ~ p21/Cdk2 (24h)	3.77 (3.08, 0.2445)	-3.32 (3.09)	0.3029	0.54 (-0.26 to 0.90, 0.1649)	0.3159
		Female		0.45 (0.10, 0.0006)			0.85 (0.35 to 0.97, 0.0081)	
6e	Human GBM	Male	SA-β-gal ~ p21 (5d)	-5.50 (31.3, 0.8636)	28.05 (31.79)	0.3949	-0.09 (-0.75 to 0.66, 0.8408)	**0.0461**
		Female		22.56 (5.51, 0.0015)			0.83 (0.29 to 0.97, 0.0115)	
6f	Human GBM	Male	SA-β-gal ~ p21/Cdk2 (5d)	-1.69 (2.96, 0.5786)	2.18 (2.96)	0.4770	-0.27 (-0.82 to 0.54, 0.5179)	**0.0192**
		Female		0.48 (0.11, 0.0011)			0.84 (0.32 to 0.97, 0.0099)	
8d (left)	FCG GBM	XY+	SA-β-gal ~ *Cdkn1a*	-3.81 (48.26, 0.9390)	N/A	N/A	-0.04 (-0.96 to 0.96, 0.9562)	N/A
		XY-		86.62 (53.83, 0.1462)			0.97 (0.11 to 1.00, 0.0315)	
		XX+		9.99 (41.46, 0.8157)			-0.12 (-0.95 to 0.97, 0.8797)	
		XX-		73.31 (58.01, 0.2419)			0.85 (-0.61 to 1.00, 0.1531)	
8d (center)	FCG GBM	XY	SA-β-gal ~ *Cdkn1a*	31.13 (33.22, 0.37)	-0.51 (42.73)	0.9908	0.36 (-0.46 to 0.85, 0.386)	0.9
		XX		30.63 (26.88, 0.28)			0.42 (-0.40 to 0.87, 0.2968)	

8d (right)	FCG GBM	Sry+	SA-β-gal ~ *Cdkn1a*	10.66 (25.02, 0.6777)	70.19 (41.51)	0.1167	0.13 (-0.63 to 0.76, 0.7617)	**0.03**
		Sry-		80.85 (33.13, 0.0311)			0.91 (0.58 to 0.98, 0.0016)	
8e (left)	FCG GBM	XY+	SA-β-gal ~ *Cdkn1a/Cdk2*	-19.29 (39.83, 0.6412)	N/A	N/A	-0.26 (-0.98 to 0.93, 0.7402)	N/A
		XY-		121.83 (75.69, 0.1462)			0.94 (-0.24 to 1.00, 0.0625)	
		XX+		-24.23 (35.11, 0.5096)			-0.33 (-0.98 to 0.92, 0.6665)	
		XX-		106.28 (79.69, 0.2190)			0.87 (-0.57 to 1.00, 0.1348)	
8e (center)	FCG GBM	XY	SA-β-gal ~ *Cdkn1a/Cdk2*	7.21 (36.51, 0.8468)	-1.56 (46.98)	0.974	0.08 (-0.66 to 0.74, 0.848)	0.9941
		XX		5.65 (29.58, 0.8518)			0.08 (-0.66 to 0.74, 0.8567)	
8e (right)	FCG GBM	Sry+	SA-β-gal ~ *Cdkn1a/Cdk2*	-16.42 (22.02, 0.4702)	131.06 (51.73)	**0.0262**	-0.22 (-0.80 to 0.57, 0.5964)	**0.01**
		Sry-		114.64 (46.81, 0.0306)			0.91 (0.55 to 0.98, 0.002)	

*N/A = Not applicable

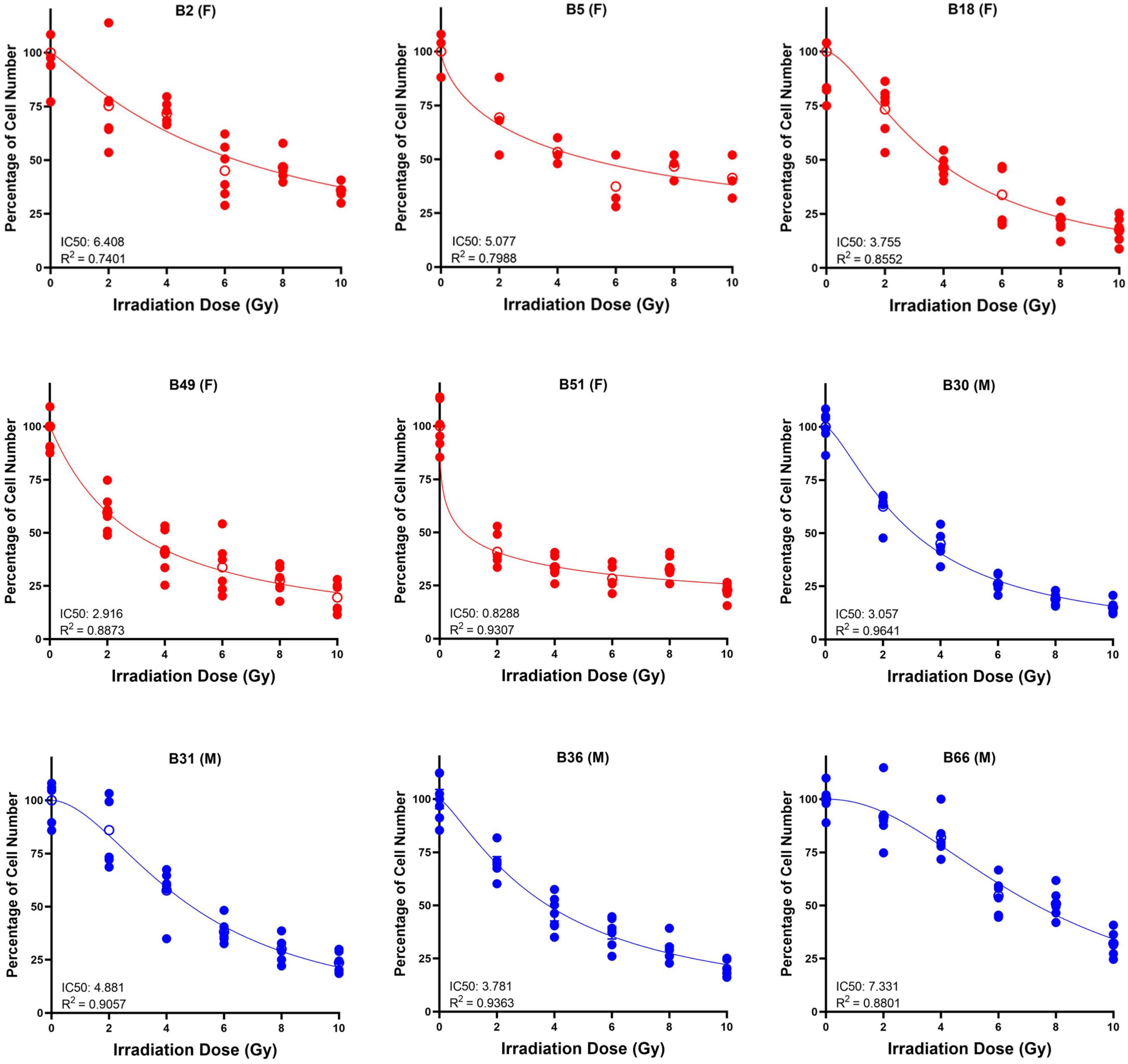

Supplementary Fig. 1: Irradiation dose response curves for male and female human GBM lines. Irradiation dose response curves for 4 male (M) and 5 female (F) primary human GBM lines. Cells were irradiated 24 hours after plating, and cell number was counted 4 days after irradiation. Percentage of cell number was calculated by dividing the cell counts for each dose by the average cell number at 0 Gy. Open symbols are means and filled symbols are the individual replicate values (B5 n=3/dose, all other lines n=6/dose). Lines are non-linear fits to the individual data points. Goodness of each fit is indicated by the R^2 value. IC_{50} values were calculated from the curve fit.

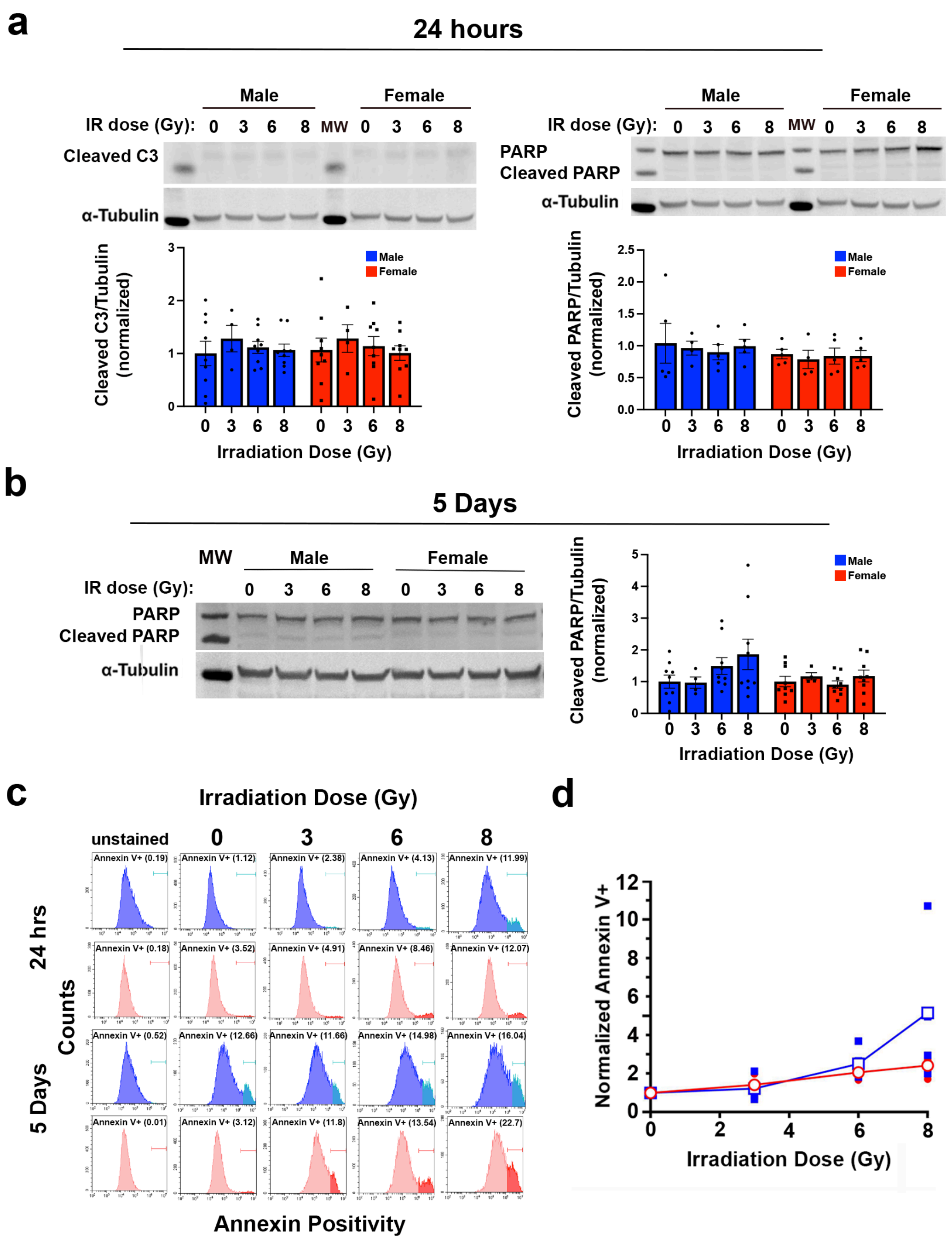

Supplementary Fig. 2: Apoptosis does not explain increased female sensitivity to radiation. a Representative western blot images and quantification of cleaved caspase-3 and cleaved PARP at 24 hours after irradiation with 0, 3, 6, or 8 Gy. Images are from one male and one female *Nf1-/- DNp53* cell line. Cleaved caspase-3 (CC3) or PARP values were first normalized to the corresponding α-tubulin. Then all CC3/α-tubulin and cleaved PARP/α-tubulin values were normalized to the corresponding Male 0 Gy condition of the same cell line, which was arbitrarily set at 1. Molecular weight markers: Cleaved caspase-3 (15 kDa), Total PARP (190 kDa), Cleaved PARP (115 kDa), α-tubulin (50 kDa). Two-way ANOVA for cleaved caspase-3 results: Dose p=0.6663, Sex p=0.9464, Interaction p=0.9887. Two-way ANOVA for cleaved PARP results: Dose p=0.9395, Sex p=0.2134, Interaction p=0.9807. Data are means +/- SEM (n=4-9/sex/dose). **b** Representative western blot images and quantification of cleaved PARP 5 days after irradiation with 0, 3, 6, or 8 Gy. Image is from one male and one female *Nf1-/- DNp53* cell line. Molecular weight markers: Total PARP (190 kDa), Cleaved PARP (115 kDa), α-tubulin (50 kDa). Two-way ANOVA Dose p=0.2099, Sex p=0.1957, Interaction p=0.3465. Data are means +/- SEM (n=4-9/sex/dose). Values (**a**, **b**) were normalized to the corresponding Male 0 Gy condition of the same cell line, which was arbitrarily set at 1. **c** Representative histograms for annexin V positivity 24 hours and 5 days after irradiation with 0, 3, 6, or 8 Gy in male (blue) and female (red) *Nf1-/- DNp53* astrocytes. **d** Quantification of annexin V positivity 24 hrs after irradiation. Values were normalized to the corresponding Male 0 Gy condition of the same cell line, which was arbitrarily set at 1. Means and connecting lines are presented as open symbols (Male – Blue, Female – Red). Individual replicate vales are shown as smaller filled symbols of the corresponding color. Two-way ANOVA Dose *p=0.0111, Sex 0.3428, Interaction 0.7077. Data are means +/- SEM (n=4/sex/dose).

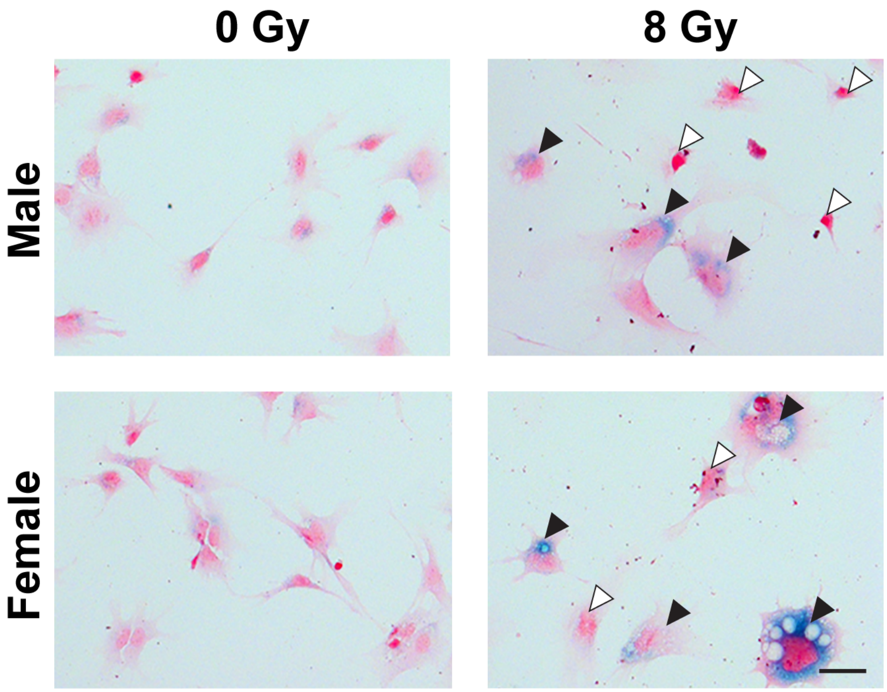

Supplementary Fig. 3: Irradiation induces changes in cell size and shape consistent with senescence. Example images of male and female *Nf1-/- DNp53* astrocytes stained for SA-β-gal (blue) 5 days after irradiation with 0 or 8 Gy, followed by counterstaining with nuclear fast red (pink). Scale bar, 50 μm. Images are magnified from Fig. 3c. Untreated male and female cells show some variation in size and morphology but are generally small with very little blue staining. Treated cells show much greater variation in size and morphology, and an increase in the percentage of cells with blue staining. Black arrowheads indicate large, irregularly shaped cells that are present after irradiation; white arrowheads indicate cells that retain normal *Nf1-/- DNp53* astrocyte morphology following irradiation.

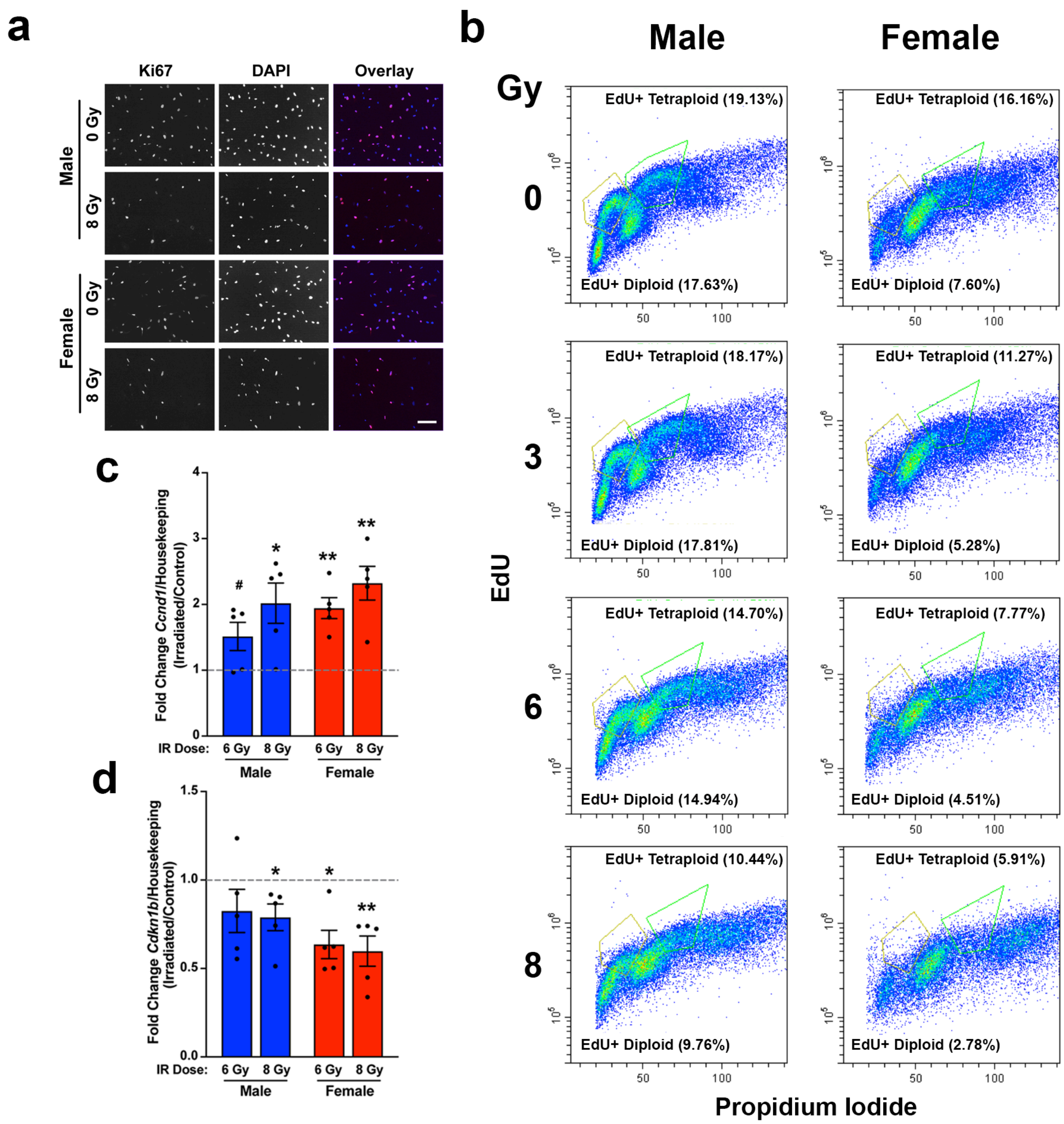

Supplementary Fig. 4: Irradiation leads to cell cycle arrest and altered expression of senescence associated genes in male and female *Nf1-/- DNp53* astrocytes. a Example immunofluorescence images of *Nf1-/- DNp53* astrocytes stained for the cell proliferation marker Ki67 5 days after irradiation with 0 or 8 Gy. Nuclei were counterstained with DAPI. Scale bar, 150 μm. **b** Example EdU incorporation density plots in response to irradiation with 0, 3, 6, or 8 Gy in male and female *Nf1-/- DNp53* astrocytes, measured 24 hours after irradiation. **c** Fold change in expression of *Ccnd1* (Cyclin D1) mRNA 5 days after irradiation with 0, 6, or 8 Gy, measured by qPCR. $^{\#}$p=0.0747, *p<0.05, **p<0.01 vs 1.0, one sample t-test. Shown are the means +/- SEM as well as the individual replicate data points (n=5/sex/dose). **d** Fold change in expression of *Cdkn1b* (p27) mRNA 5 days after irradiation with 0, 6, or 8 Gy, measured by qPCR. *p<0.05, **p<0.01 vs 1.0, one sample t-test. Shown are the means +/- SEM as well as the individual replicate data points (n=5/sex/dose).

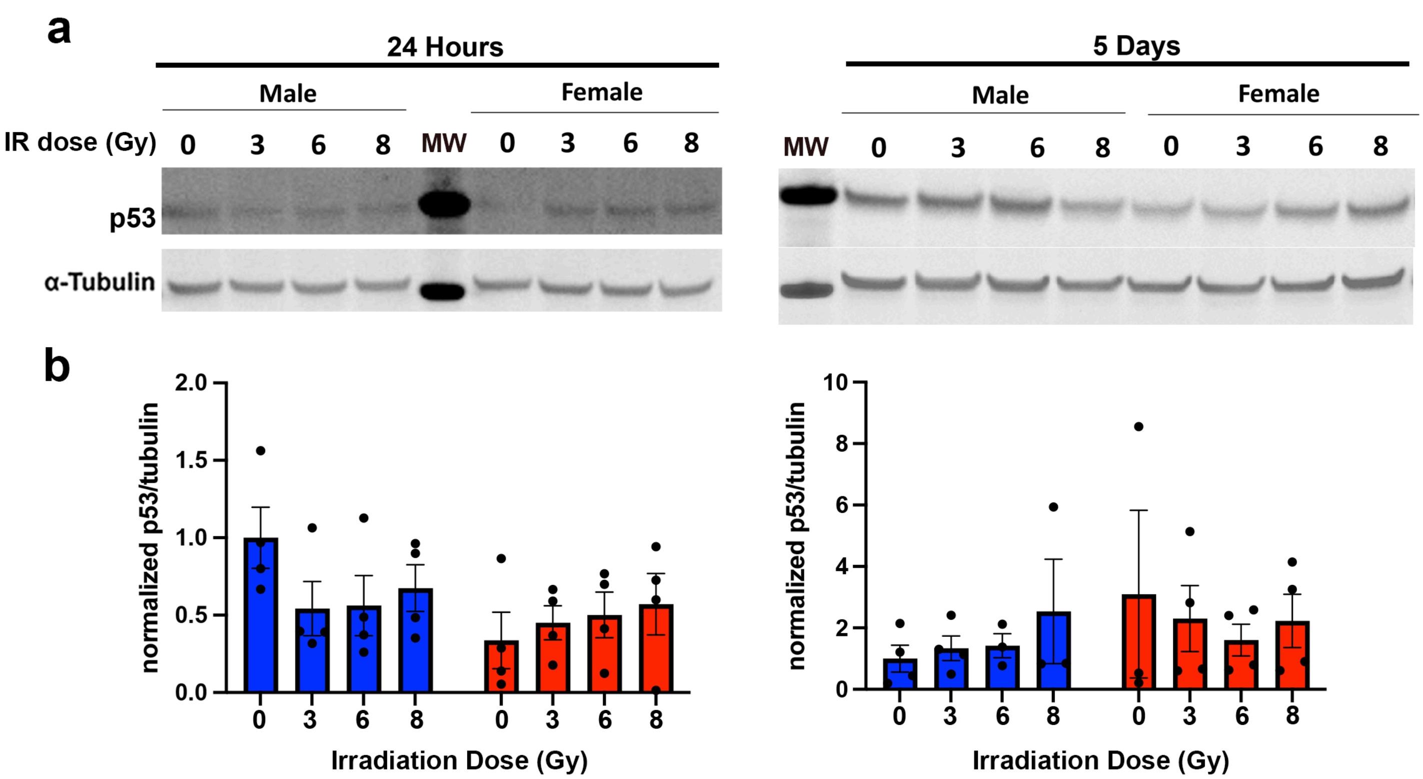

Supplementary Fig. 5: Endogenous p53 expression is not induced by irradiation of *Nf1-/- DNp53* astrocytes. a Representative western blot image of endogenous p53 expression in male and female *Nf1-/- DNp53* astrocytes 24 hours and 5 days after irradiation with 0, 3, 6, or 8 Gy. **b** Quantification of p53 levels measured by western blot. p53 values were first normalized to the corresponding α-tubulin. Then all p53/α-tubulin values were normalized to the corresponding Male 0 Gy condition of the same cell line, which was arbitrarily set at 1. Molecular weight markers: p53 (50 kDa), α-tubulin (50 kDA). Two-way ANOVA for p53 24 hrs: Dose p=0.7357, Sex p=0. 0709, Interaction p=0.2630. Two-way ANOVA for p53 5 days: Dose p=0.8889, Sex p=0.3633, Interaction p=0.7361. Shown are the means +/- SEM as well as the individual replicate data points (n=4/sex/dose)

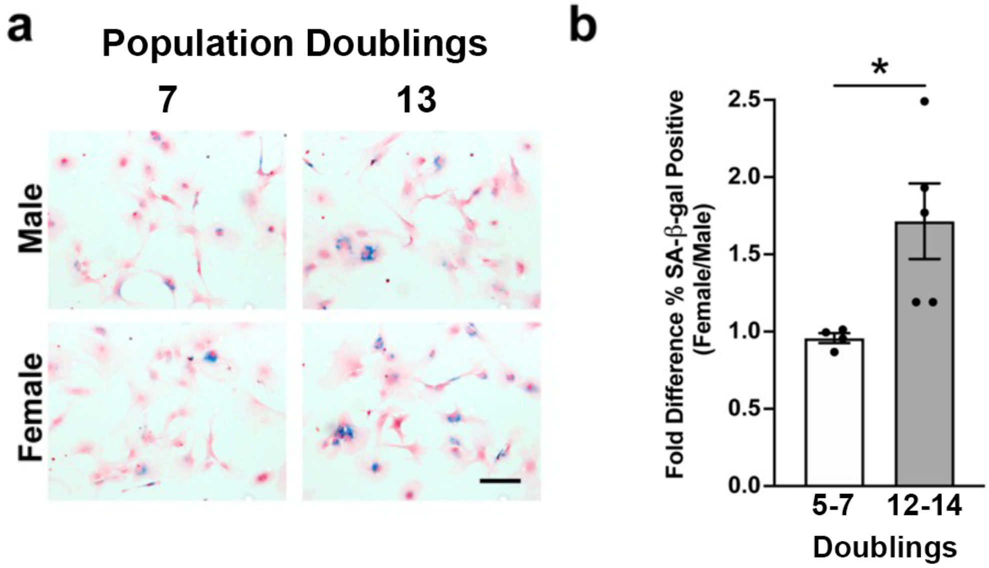

Supplementary Fig. 6: Female wildtype astrocytes have increased senescent cells with advanced population doublings. a Example images of male and female wildtype mouse astrocytes at low (7) or high (13) numbers of population doublings stained for SA-β-gal and counterstained with nuclear fast red. Scale bar, 150 μm. **b** Fold difference in the percentage of SA-β-gal positive cells in male and female cultures at either low (5-7) or high (12-14) population doublings. *p<0.05, two-tailed t-test with Welch's correction. Shown are the means +/- SEM as well as the individual replicate data points (n=4-5/group).

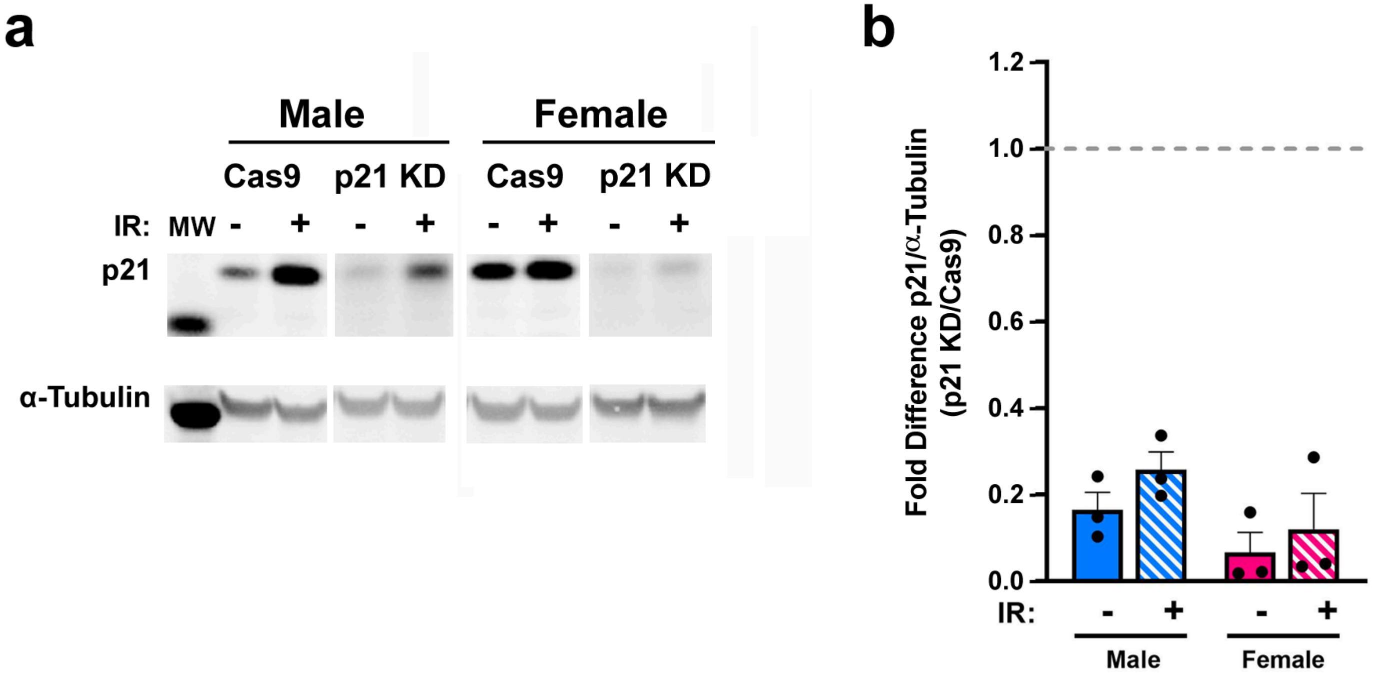

Supplementary Fig. 7: Confirmation of p21 knockdown. a Western blot images of p21 levels in *Nf1-/- DNp53* Cas9 control and p21 knockdown male and female astrocytes 24 hours after irradiation with 0 or 8 Gy. Molecular weight markers: p21 (15 kDa), α-tubulin (50 kDa). **b** Fold knockdown in p21 was calculated by dividing the levels of p21 expression in p21 KD lines by the levels in Cas9 control lines under non-irradiated and irradiated conditions. Shown are the means and SEM as well as individual replicate points (n=3). There were no significant differences detected by One-Way ANOVA and Sidak's multiple comparisons test.

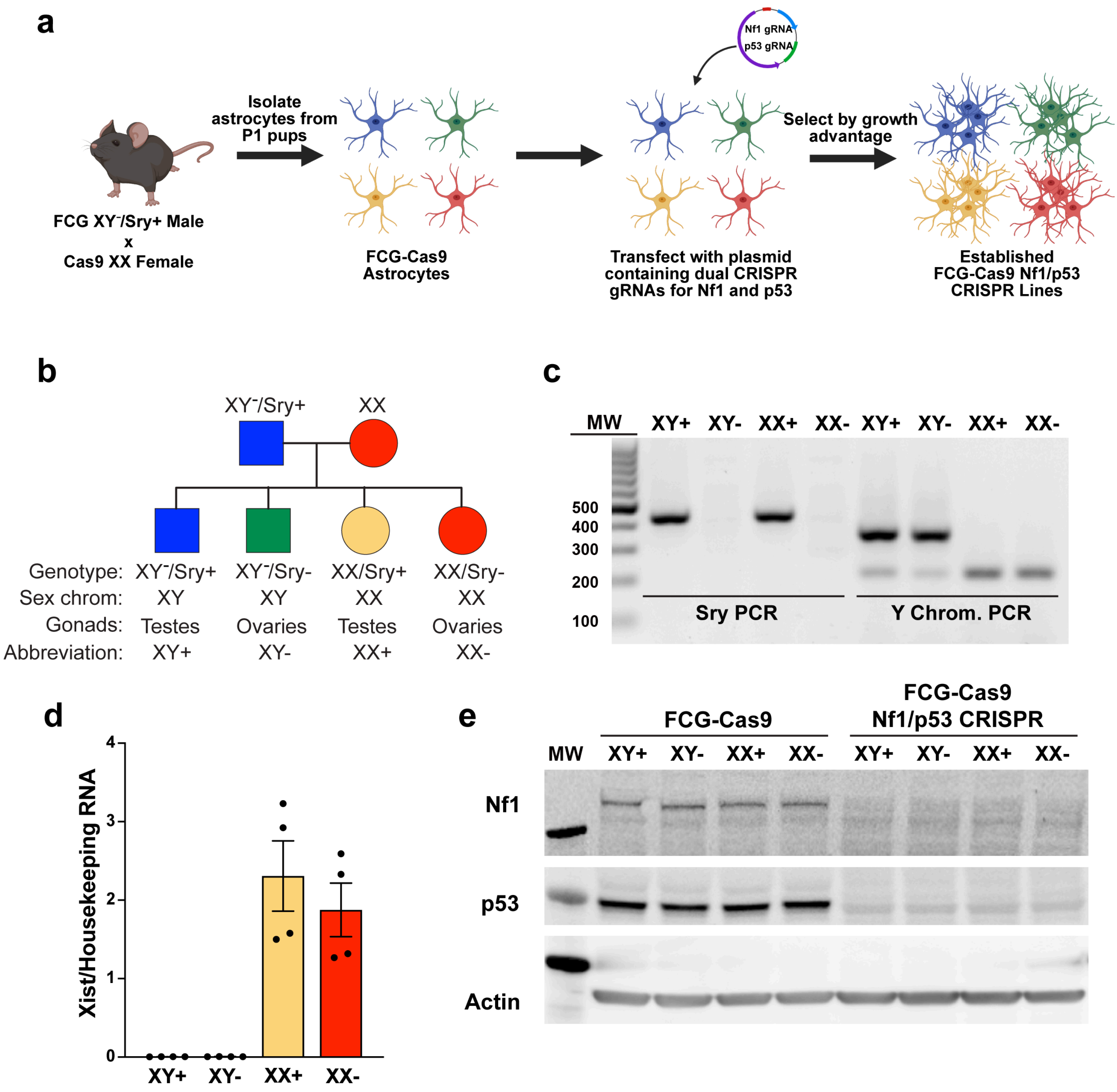

Supplementary Fig. 8: Four Core Genotypes model of GBM. **a** Schematic for generation of the FCG GBM model (created with BioRender.com). **b** Diagram of the four genotypes resulting from the FCG mouse model. **c** Genotyping PCR (Sry and Y chromosome) confirming correct genotypes in astrocyte cultures isolated from FCG-Cas9 postnatal day 1 pups. PCR product size is indicated by DNA ladder with corresponding base-pair sizes. **d** Expression levels of the lncRNA Xist, measured by qPCR, in FCG-Cas9 Nf1/p53 CRISPR astrocytes, showing appropriate expression in XX cells only, regardless of gonadal sex. Results are from 2 cell lines, with 2 technical replicates each. Shown are means and SEM as well as individual replicate points (n=4/genotype). **e** Western blot images of Nf1 and p53 expression in FCG-Cas9 and FCG-Cas9 Nf1/p53 CRISPR astrocytes, confirming knockdown of these two proteins in the FCG GBM model. Molecular weight markers: Nf1 (190 kDa), p53 (50 kDa), actin (50 kDa).

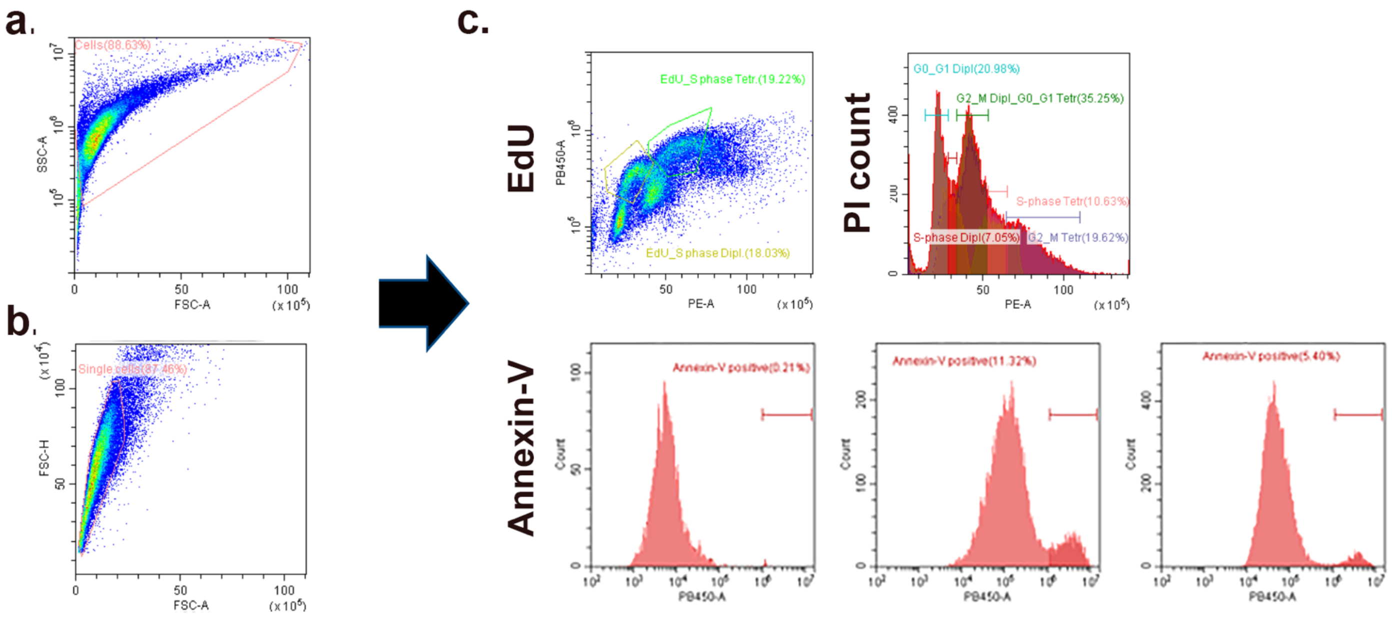

Supplementary Fig. 9: Gating strategy for EdU and Annexin-V flow cytometry. **a**. Forward (FSCA) and side (SSC-A) scatter of Nf1-/- DNp53 cells with gate for single cells. **b**. FSC-A and FSC-H scatter with single cells identified. c. Single cells were then utilized in EdU, Propidium iodide and Annexin-V analysis as shown. Example gating is shown for EdU and Propidium iodide (top row) and Annexin-V(left to right: unstained (negative control), etoposide-treated (positive control) and control culture conditions).
