# Supplementary material for: Gonadal sex patterns p21-induced cellular senescence in mouse and human glioblastoma
Source: Commun Biol. 2022 Aug 2;5:781. doi: 10.1038/s42003-022-03743-9 (PMC9345919; doi:10.1038/s42003-022-03743-9)
Supplement: Supplementary file 3 — Description of Additional Supplementary Files [file 42003_2022_3743_MOESM3_ESM.pdf]

Description of Additional Supplementary Files

File name: Supplementary Data
Description: The source data for all figures and tables and uncropped and unedited western blot images.
