# Supplementary material for: Gonadal sex patterns p21-induced cellular senescence in mouse and human glioblastoma
Source: Commun Biol. 2022 Aug 2;5:781. doi: 10.1038/s42003-022-03743-9 (PMC9345919; doi:10.1038/s42003-022-03743-9)
Supplement: Supplementary file 5 — Reporting Summary [file 42003_2022_3743_MOESM5_ESM.pdf]

nature research

Corresponding author(s): Joshua B Rubin

Last updated by author(s): 05.15.2022

Reporting Summary

Nature Research wishes to improve the reproducibility of the work that we publish. This form provides structure for consistency and transparency in reporting. For further information on Nature Research policies, see our Editorial Policies and the Editorial Policy Checklist.

Statistics

For all statistical analyses, confirm that the following items are present in the figure legend, table legend, main text, or Methods section.

n/a	Confirmed	
☐	☒	The exact sample size (n) for each experimental group/condition, given as a discrete number and unit of measurement
☐	☒	A statement on whether measurements were taken from distinct samples or whether the same sample was measured repeatedly
☐	☒	The statistical test(s) used AND whether they are one- or two-sided<br>*Only common tests should be described solely by name; describe more complex techniques in the Methods section.*
☒	☐	A description of all covariates tested
☐	☒	A description of any assumptions or corrections, such as tests of normality and adjustment for multiple comparisons
☐	☒	A full description of the statistical parameters including central tendency (e.g. means) or other basic estimates (e.g. regression coefficient) AND variation (e.g. standard deviation) or associated estimates of uncertainty (e.g. confidence intervals)
☐	☒	For null hypothesis testing, the test statistic (e.g. F, t, r) with confidence intervals, effect sizes, degrees of freedom and P value noted<br>*Give P values as exact values whenever suitable.*
☒	☐	For Bayesian analysis, information on the choice of priors and Markov chain Monte Carlo settings
☒	☐	For hierarchical and complex designs, identification of the appropriate level for tests and full reporting of outcomes
☒	☐	Estimates of effect sizes (e.g. Cohen's d, Pearson's r), indicating how they were calculated

Our web collection on statistics for biologists contains articles on many of the points above.

Software and code

Policy information about availability of computer code

Data collection

Western blot band intensity was quantified using Image Lab Software Version 6.0.0 (Bio-Rad)

For SA-beta-gal quantification, total cells and positive cells were counted by hand in ImageJ (FIJI Version 2.1.0/1.53c) using the Cell Counter plugin.

For Ki67 quantification, color images were converted to 16bit and thresholded in ImageJ (Version 1.53e with Java 1.8.0_172 [64 bit]). After thresholding, cell number was counted with the automated cell counter (Analyze Particles); anything <60 pixel2 was excluded from the count.

Clonogenic assay plate images were analyzed with ImageJ (Java 1.8.0_172), utilizing ImageJ's thresholding, ROI (region of interest) manager, and analyze particles functions to count individual colonies.

For qPCR analysis, a CFX Connect Real-Time PCR Detection System (Bio-Rad) was used to collect and analyze the amplification data.

For live cell imaging, cell confluence was analyzed using the IncuCyte ZOOM analysis software (Sartorius).

Data analysis

Statistics: Data were graphed and statistical analyses, including two-way ANOVAs, t-tests, one sample t-tests and dose response curves, were performed using GraphPad Prism software (Prism 9 for macOS Version 9.0.0). All tests were two-tailed unless otherwise specified. The two-way ANOVA models fit the effect of sex, time or dosage, and their interaction, followed by post-hoc multiple comparisons using the Bonferroni correction. Dose response curves were fit to normalized response versus dosage using the 4-parameter logistic regression dose-response model with the slopes estimated by maximizing the likelihood function. Spearman rank-based correlation analysis between IC50 and gene expression was performed as described using R (version 3.5.0). Briefly, Spearman correlation coefficients of irradiation IC50 values with expression of either MC5 genes (17 genes), FC3 genes (9 genes), or randomly selected gene sets of the same size, were calculated. The Olkin-averaged Spearman correlation coefficients with IC50 across MC5 genes and across FC3 genes per cell line was summarized and tested against 0 with those derived for 1000 random gene sets serving as negative control. To explore the relationship between senescence and the

various molecular markers focused on p16, p21, and p21/CDK2 ratio, linear regression analysis and Pearson's correlation analysis was conducted using R (version 4.0.2). Pearson's correlation coefficients (r) were calculated and the Fisher's Z transformed coefficients were compared between sex by normal test. In the integrative linear regression modeling across sex, the dependent/outcome variable was senescence, as measured by SA-beta-gal, while the independent/predictor variable includes the continuous mRNA or protein levels of the molecular marker of interest and sex. To assess the possible linear association between SA-beta-gal and each of the continuous variables by sex, sex-specific intercepts and slopes were estimated from the integrative linear models. The slope difference between the male and female-specific slopes was derived with 95% confidence interval (CI) and tested against 0 by the 2-sided Wald test. Significance was claimed at $p<0.05$.

For manuscripts utilizing custom algorithms or software that are central to the research but not yet described in published literature, software must be made available to editors and reviewers. We strongly encourage code deposition in a community repository (e.g. GitHub). See the Nature Research guidelines for submitting code & software for further information.

Data

Policy information about availability of data

All manuscripts must include a data availability statement. This statement should provide the following information, where applicable:

- Accession codes, unique identifiers, or web links for publicly available datasets
- A list of figures that have associated raw data
- A description of any restrictions on data availability

The data that support the findings of this study are available from the corresponding author upon reasonable request.

Field-specific reporting

Please select the one below that is the best fit for your research. If you are not sure, read the appropriate sections before making your selection.

☒ Life sciences ☐ Behavioural & social sciences ☐ Ecological, evolutionary & environmental sciences

For a reference copy of the document with all sections, see nature.com/documents/nr-reporting-summary-flat.pdf

Life sciences study design

All studies must disclose on these points even when the disclosure is negative.

Sample size	No sample size calculations were performed. All experiments consisted of sample sizes greater than or equal to 3 biological replicates.
Data exclusions	No data points were excluded.
Replication	All experiments consisted of sample sizes greater than or equal to 3 biological replicates. Key findings were observed across mutiple cell lines and models.
Randomization	No experiments required randomization.
Blinding	All images were taken and quantified by an experimenter blinded to the sex and experimental condition of the sample.

Reporting for specific materials, systems and methods

We require information from authors about some types of materials, experimental systems and methods used in many studies. Here, indicate whether each material, system or method listed is relevant to your study. If you are not sure if a list item applies to your research, read the appropriate section before selecting a response.

Materials & experimental systems

n/a	Involved in the study	
☐	☒	Antibodies
☐	☒	Eukaryotic cell lines
☒	☐	Palaeontology and archaeology
☐	☒	Animals and other organisms
☐	☒	Human research participants
☒	☐	Clinical data
☒	☐	Dual use research of concern

Methods

n/a	Involved in the study	
☒	☐	ChIP-seq
☐	☒	Flow cytometry
☒	☐	MRI-based neuroimaging

Antibodies

Antibodies used: 1:1000 anti-cleaved caspase-3 Cell Signaling Technologies #9664, 1:4000 anti-alpha-Tubulin Sigma #T5168, 1:1000 anti-PARP Cell

Antibodies used

Signaling Technologies #9542, 1:2000 anti-mouse p21 abcam #ab188224, 1:1000 anti-Cdk2 Cell Signaling Technologies #2546, 1:1000 anti-human p21 Cell Signaling Technologies #2947, 1:200 anti-Nf1 Santa Cruz #sc-376886, 1:2000 anti-p53 Leica #NCL-L-p53-CM5p, 1:25,000 anti-Actin Sigma #A1978, γH2AX (Millipore, 05–636 JBW301), Fluor 488 goat anti-mouse IgG (Invitrogen, A28181), Cleaved caspase-3 (Cell Signaling Technologies #9664), Beta-Tubulin (Sigma #T5168), PARP (Cell Signaling Technologies #9542), mouse p21 (Abcam #ab188224), Cdk2 (Cell Signaling Technologies #2546), human p21 (Cell Signaling Technologies #2947), Nf1 (Santa Cruz #sc-376886), p53 (Sigma #T5168), Actin (Sigma #A1978), IRDye 680RD Donkey anti-Rabbit, Donkey anti-mouse, IRDye 800CW Donkey anti-Rabbit, Donkey anti-mouse (LICOR), Annexin-V (Pacific Blue, Invitrogen, A35122), Ki67 (Abcam ab15580), donkey anti-rabbit Alexa Fluor 555 (Invitrogen a31572), geminin (Abcam ab175799)

Validation

All antibodies were commercially available and validated by the vendor.

Eukaryotic cell lines

Policy information about cell lines

Cell line source(s)

Human GBM Cell Lines - provided by Dr. Albert H. Kim (Washington University School of Medicine)
Nf1-/- DNp53 Mouse GBM model lines - developed in the Joshua B. Rubin lab (Washington University School of Medicine)
Nf1-/- DNp53 Cas9 and p21 KD lines - developed in the Joshua B. Rubin lab (Washington University School of Medicine)
FCG-Cas9 Nf1/p53 CRISPR mouse lines - developed in the Joshua B. Rubin lab (Washington University School of Medicine)

Authentication

All murine cell lines were primary cell lines. Identification of astrocyte lineage was confirmed by immunocytochemistry for GFAP (positive), neurofilament (negative), CNPase (negative). Sex was confirmed by PCR genotyping. Knockdowns were confirmed by Western Blot. Patient derived cell lines were confirmed by DNA sequencing and RNA expression.

Mycoplasma contamination

Primary cell lines were not tested for mycoplasma contamination

Commonly misidentified lines
(See ICLAC register)

N/A

Animals and other organisms

Policy information about studies involving animals; ARRIVE guidelines recommended for reporting animal research

Laboratory animals

All astrocytes used in the study were derived from postnatal day 1 male and female (or in the case of the Four Core Genotypes model XY/Sry+, XY/Sry-, XX/Sry+, and XX/Sry-) mouse pups on a C57Bl6/J background. Male and female astrocytes were analyzed separately.

Wild animals

N/A

Field-collected samples

N/A

Ethics oversight

All animals were used in accordance with an Animal Studies Protocol approved by the Animal Studies Committee of the Washington University School of Medicine, per the recommendations of the Guide for the Care and Use of Laboratory Animals (NIH).

Note that full information on the approval of the study protocol must also be provided in the manuscript.

Human research participants

Policy information about studies involving human research participants

Population characteristics

The patient population were adults requiring neurosurgical management of newly diagnosed glioblastoma. There was no restriction based on sex, gender, or race. By necessity all patients were older than 21.

Recruitment

Patients were approached for consent to use their tumor tissue for derivation of cell lines in an adult Neurosurgical practice without regard to sex, gender, or race.

Ethics oversight

Consent was obtained in accordance with a Washington University Institutional Review Board (IRB) approved Human Studies Protocol.

Note that full information on the approval of the study protocol must also be provided in the manuscript.

Flow Cytometry

Plots

Confirm that:

☒ The axis labels state the marker and fluorochrome used (e.g. CD4-FITC).

☒ The axis scales are clearly visible. Include numbers along axes only for bottom left plot of group (a 'group' is an analysis of identical markers).

☒ All plots are contour plots with outliers or pseudocolor plots.

☒ A numerical value for number of cells or percentage (with statistics) is provided.

Methodology

Sample preparation

Murine astrocytes were seeded at a density of 2x105 (females) and 1x105 (males) in T25 flasks and cultured in DMEM/F12 with 10% FBS and 1% penicillin-streptomycin. The next day cells were treated with 0, 3, 6 or 8 Gy irradiation (IR) and returned to the incubator. Cell cycle analysis was conducted at 24 hours after irradiation. For annexin-V staining, cells were labelled with 5 l Annexin-V (Pacific Blue, Invitrogen, A35122) in 100 L 1X Annexin-V binding buffer (Invitrogen, V13246) for 15 minutes in the dark at room temperature. After 15 minutes incubation, 400 l of 1X Annexin-V binding buffer was added to each sample and FACS analyzed using the CytoFlex S Flow Cytometer (Beckman Coulter, CO2949). Etoposide (final concentration of 20 g) was used as a positive indicator of apoptosis. Edu-PI: For EdU-PI staining, cells were prepared and resuspended in 1X Click-iTTM permeabilization and wash reagent per the manufacturer's instructions (Invitrogen, C10636). Detection of Click-iT EdU was followed immediately by incubating the cells with the Click-iT Plus reaction cocktail (438 L PBS, 10 L copper protectant, 2.5 L fluorescent dye picolyl azide, 50 L reaction buffer additive per sample) for 30 minutes at room temperature, protected from light. Cells were then washed with 3 mL 1X Click-iTTM permeabilization and wash reagent, centrifuged, and resuspended in 200 L of 1X Click-iT permeabilization and wash reagent containing 0.3 L PI, and FACS analyzed using the CytoFlex S Flow Cytometer (Beckman Coulter, CO2949).

Instrument

CytoFlex S Flow Cytometer (Beckman Coulter, CO2949)

Software

CytExpert 2.3 software

Cell population abundance

The abundance of apoptotic cells was determined from a single parameter histogram. The gate for annexin-V positivity was determined by using unstained cells and cells treated with Etoposide as negative and positive controls, respectively. The fraction of apoptotic cells was calculated from the apoptotic cell number divided by the total number of events analyzed.The proportion of the cells in the 2N and 4N S-phases were determined from two parameter histogram tained cells and cells treated with Etoposide as negative and positive controls, respectively. For cell cycle analysis, gates were drawn using the EdU-positive (Pacific Blue, PB450-A) versus PI-positive (PE-A) channels to determine the percentage of cells in S-phase, using 48-hour starved cells as negative controls.

Gating strategy

Cells were first gated on the FSC-A versus SCC-A plot to exclude the debris/cell fragments and pyknotic cells in the bottom left corner. This was followed by gating cells in the FSC-H versus FSC-A plot to gate onto the single-cell population and exclude the doublets or cellular clumps from the analysis. For Apoptosis analysis, a single parameter histogram was used to gate onto the Annexin-V positive cells using unstained cells and cells treated with Etoposide as negative and positive controls, respectively. For cell cycle analysis, gates were drawn using the EdU-positive (Pacific Blue, PB450-A) versus PI-positive (PE-A) channels to determine the percentage of cells in S-phase, using 48-hour starved cells as negative controls. Gating parameters remained consistent across all treatment groups.

☒ Tick this box to confirm that a figure exemplifying the gating strategy is provided in the Supplementary Information.
